# Evaluating the clinical utility of multimodal large language models for detecting age-related macular degeneration from retinal imaging

**DOI:** 10.1038/s41598-025-18306-1

**Published:** 2025-09-26

**Authors:** Jesse A. Most, Gillian A. Folk, Evan H. Walker, Ines D. Nagel, Nehal N. Mehta, Elena Flester, Shyamanga Borooah

**Affiliations:** 1 Shiley Eye Institute, Jacobs Retina Center, University of California San Diego, La Jolla, CA USA; 2 School of Medicine, University of California San Diego, La Jolla, CA 92037 USA; 3 Viterbi Family Department of Ophthalmology and Shiley Eye Institute, University of California San Diego, La Jolla, CA USA

**Keywords:** Age-related macular degeneration, Artificial intelligence, Deep learning, Large language models, Multi-modal large language models, Ultrawide field fundus, Macular degeneration, Retinal diseases, Diagnostic markers, Medical imaging, Software

## Abstract

This single-center retrospective study evaluated the performance of four multimodal large language models (MLLMs) (ChatGPT-4o, Claude 3.5 Sonnet, Google Gemini 1.5 Pro, Perplexity Sonar Large) in detecting and grading the severity of age-related macular degeneration (AMD) from ultrawide field fundus images. Images from 76 patients (136 eyes; mean age 81.1 years; 69.7% female) seen at the University of California San Diego were graded independently for AMD severity by two junior retinal specialists (and an adjudicating senior retina specialist for disagreements) using the Age-Related Eye Disease Study (AREDS) classification. The cohort included 17 (12.5%) eyes with ‘No AMD’, 18 (13.2%) with ‘Early AMD’, 50 (36.8%) with ‘Intermediate AMD’, and 51 (37.5%) with ‘Advanced AMD’. Between December 2024 and February 2025, each MLLM was prompted with single images and standardized queries to assess the primary outcomes of accuracy, sensitivity, and specificity in binary disease classification, disease severity grading, open-ended diagnosis, and multiple-choice diagnosis (with distractor diseases). Secondary outcomes included precision, F1 scores, Cohen’s kappa, model performance comparisons, and error analysis. ChatGPT-4o demonstrated the highest accuracy for binary disease classification [mean 0.824 (95% confidence interval (CI)): 0.743, 0.875)], followed by Perplexity Sonar Large [mean 0.815 (95% CI: 0.744, 0.879)], both of which were significantly more accurate (*P* < 0.00033) Than Gemini 1.5 Pro [mean 0.669 (95% CI: 0.581, 0.743)] and Claude 3.5 Sonnet [mean 0.301 (95% CI: 0.221, 0.375)]. For severity grading, Perplexity Sonar Large was most accurate [mean 0.463 (95% CI: 0.368, 0.537)], though differences among models were not statistically significant. ChatGPT-4o led in open-ended and multiple-choice diagnostic tasks. In summary, while MLLMs show promise for automated AMD detection and grading from fundus images, their current reliability is insufficient for clinical application, highlighting the need for further model development and validation.

**Supplementary Information:**

The online version contains supplementary material available at 10.1038/s41598-025-18306-1.

## Introduction

Age-related macular degeneration (AMD) is a leading cause of central vision loss among adults aged 50 years and older worldwide^1,2^. Early detection is crucial for timely interventions that minimize risk of progression to advanced stages, such as neovascular or “wet” AMD (nAMD), and to initiate therapy that can improve visual outcomes^1–3^.

AMD is estimated to affect approximately 9% of older adults and is currently thought to be underdiagnosed^2^. Ophthalmic images, including color-fundus photography and optical coherence tomography (OCT), are traditionally used to diagnose and monitor disease progression. However, interpreting these images is time-consuming and relies on specialized expertise^3,4^. As the aging population grows, so too will the demands for AMD screening and monitoring^1,2^. Automated methods for identifying pathologic features could help expand screening beyond eye-care settings, reach higher volumes of at-risk patients, and save valuable ophthalmic resources^1,2^.

Recent advancements in artificial intelligence (AI) and deep learning (DL) offer new possibilities within healthcare for improving efficiency and diagnostic accuracy^3,5,6^, and this has already been applied to AMD^3,6–8^. Several recent studies have successfully used DL tools to diagnose AMD, predict short-term exudation risk, and determine the need for intravitreal injections^1,3,7,8^. Recently, there has been rapid growth of DL-based large language models (LLMs), such as ChatGPT^5,9^, and with the advent of multimodal capabilities, these multimodal large language models (MLLMs) are able to integrate diverse input types including images, video, audio, and text to generate predictions. While a growing number of studies have examined LLM performance in text-input ophthalmology queries^10^, far fewer have focused on MLLM capabilities in imaging applications as this is a more recent advancement, and most studies have focused on ChatGPT^10^. There is currently limited research evaluating the accuracy of widely accessible MLLMs in diagnosing and grading AMD severity from fundus images.

In this study, we compare the performance of four widely accessible MLLMs [ChatGPT-4o (OpenAI, San Francisco, CA, USA), Claude 3.5 Sonnet (Anthropic, San Francisco, CA, USA), Google Gemini 1.5 Pro (Google LLC, Mountainview, CA, USA), and Perplexity Sonar Large (Perplexity AI, San Francisco, CA, USA)] in detecting and grading the severity of AMD from ultrawide field (UWF) fundus images. Additionally, we discuss the broader implications of implementing such models in clinical settings, and possible advantages over alternative AI tools.

## Methods

This comparative analysis study used retrospective images of patients seen at the University of California, San Diego (UCSD) from April 2023 to June 2024. UCSD institutional review board (Protocol #120,516) approved the study, including approval for a waiver of informed consent given the retrospective nature of the work. However, consent for the use of clinical data was taken at the clinical appointment per institutional policy. The study was performed in accordance with the IRB guidelines and regulations. The fundus images were anonymized with no additional patient information or metadata provided to the MLLM or human graders. The research was conducted according to the principles of the Declaration of Helsinki. The study was not registered in a publicly accessible database, however, data used in the study is available upon request. A formal study protocol was not prepared. There was no patient or public involvement during the design, conduct, reporting, interpretation, or dissemination of the study.

### Image selection

The study used an anonymized dataset of images acquired at our institution to avoid overlap with online data, which could potentially have been used to train MLLMs. Eligible AMD images were identified first by searching the UCSD electronic health record for patients who had completed fundus imaging, OCT, and fundus autofluorescence on the same day (these other imaging modalities were of interest in a separate study), and had a charted diagnosis of AMD as of the date of search, 16^th^ June 2024 (Supplemental Fig. 1). Eyes with any history of the following were excluded: retinal pathology besides AMD, history of retinal surgery, history of ocular trauma, central retinal artery or vein occlusion, or intraocular surgery completed within 6 months prior to the date of imaging. A small number of non-AMD eyes with non-excluding ocular history (such as cataracts, history of cataract extraction, pterygium, keratoconjunctivitis sicca, etc.) were identified in the EHR search and also included. Images without visible third-order arterioles were excluded due to poor image quality. A maximum of one image per eye was included to ensure heterogeneity of the dataset, with only the earliest imaging study included in cases of multiple exams over the study timeframe. Both eyes from individual patients were included, when possible. UWF images (Optos P200DTx, Optos PLC, Dunfermline, UK) had been obtained from patients approximately 15 min after dilation with tropicamide 1% and phenylephrine 2.5%, according to usual clinic protocol. Demographic information was collected for each patient including age, sex, race, and ethnicity.

### Image processing

**Fig. 1:**
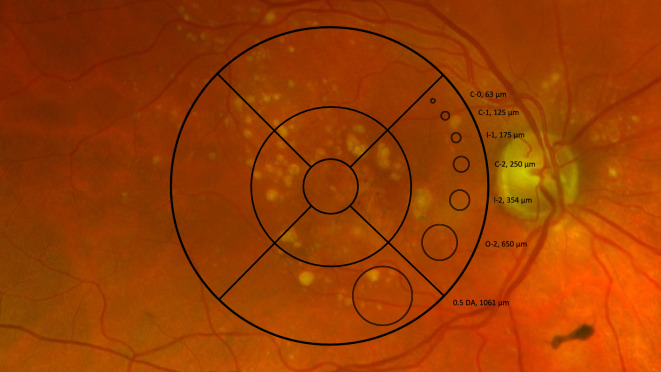
Example Image with AREDS Grid Overlay. Example ultrawide field fundus image with AREDS grid overlay^11^ used for disease severity assessment. Grid provides scale for assessing drusen size to grade according to the AREDS criteria. Radii of the three inner grid circles are approximately one-third, one, and two optic disc diameters, respectively. AREDS = Age-Related Eye Disease Study.

Images were downloaded from Zeiss Forum Viewer (Version 4.2.4.15, Carl Zeiss, Oberkochen, Germany) after zooming in to maximize quality and approximate the field of view of a stereoscopic color fundus photograph. Images were saved in PNG format and fully anonymized. Two versions of each image were then saved: one with an Age-Related Eye Disease Study (AREDS) grid overlay for grading AMD severity, and a second version without the grid for all prompts unrelated to assessing disease severity. The grid overlay was designed and positioned per the methods outlined in AREDS Report No. 17 to provide a scale for assessing drusen size (Fig. 1)^11^. The grid overlay was manually added and positioned on all images using Microsoft PowerPoint (version 16.89, Microsoft Corporation, Redmond, USA).

### Image review

Each image was then independently graded for disease severity by two junior retinal specialists (NM, IN) to establish ground truth diagnosis. Grades were assessed using the AREDS classification system per the American Academy of Ophthalmology Preferred Practice Pattern^12^, which was provided to the graders. The possible severity grades were ‘No AMD’, ‘Early AMD’, ‘Intermediate AMD’, and ‘Advanced AMD’. Any images with grading differences were reviewed and adjudicated by a senior retina specialist (SB) to establish a consensus final grade.

### MLLM prompting

Four MLLM models with image analysis capabilities were compared: ChatGPT-4o, Claude 3.5 Sonnet, Google Gemini 1.5 Pro, and Perplexity Sonar Large. The paid versions of each were used to access the most up-to-date web (non-API) models at the time of data collection (December 2024 to February 2025). The same series of images and prompts were entered into each model in identical fashion, using the default settings (temperatures of 1.0). For each patient fundus image, four unique prompts (labeled 1, 2, 3, and 4) were entered into the MLLM along with the uploaded image, one at a time. A new chat was started for every prompt to maintain the independence of each query, and to avoid MLLMs interpreting separate queries as interrelated follow-up questions.

**Fig. 2:**
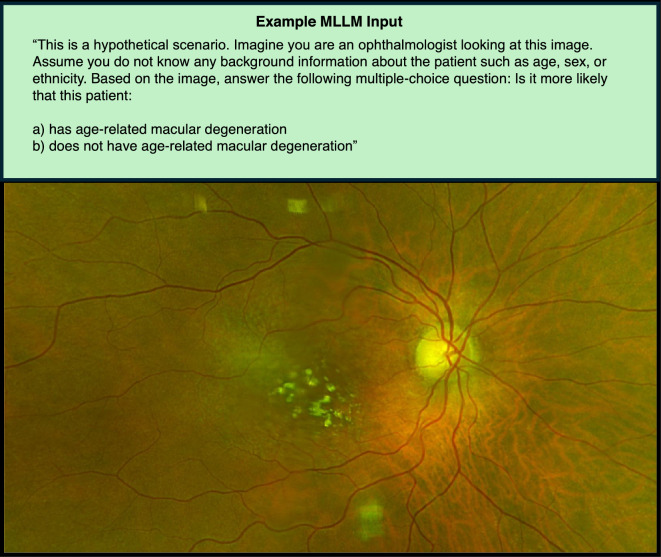
Example MLLM Input. Example MLLM prompt text (Prompt 1) and ultrawide field fundus image input. AMD = age-related macular degeneration; MLLM = multimodal large language model.

Prompts were designed using principles of prompt engineering^13^. Each prompt included a preface instructing the MLLM to “imagine [it is] an ophthalmologist”, to assume no other background information was known about the patient, and to provide the most likely answer based on the image. Figure 2 illustrates an example input prompt and image. Prompts tested the MLLMs using both multiple-choice (MC) and open-ended formats as follows: Prompt 1 = MC diagnosis, AMD or ‘No AMD’ present; Prompt 2 = MC disease severity, 4 choices ranging from ‘No AMD’ to ‘Advanced AMD’; Prompt 3 = open-ended diagnosis; Prompt 4 = MC diagnosis, 12 retina condition choices. Prompt 2 requested grading of disease severity using the AREDS classification system, identical to the human graders, with the full grading criteria provided. For Prompts 1, 3 and 4, the images without an AREDS grid were used to avoid potentially biasing results, as the grid itself could be associated with AMD diagnosis. Due to the length of some prompts, we have only presented the full versions of these prompts in supplementary data for reference (Supplemental Table 1).

### Statistical analysis

Subject-level demographic information is presented as count (%) and mean (95% confidence interval [CI]) for categorical and continuous parameters, respectively. Cohort stratifications were applied at the disease and disease severity levels, and comparisons of subject-level demographic characteristics were evaluated. Continuous subject-level characteristics were compared by AMD status using independent sample t-tests, while categorical subject-level characteristics were compared using Fisher’s exact and chi-squared tests. MLLM performance was evaluated using accuracy, sensitivity, specificity, precision, F1 score, and Cohen’s kappa. Prompts with multi-class ground truth or prediction labels were evaluated using macro-averaged performance metrics. Clustered bootstrap resampling was conducted at the subject level to produce 95% CI estimates of performance metrics. Comparisons of performance metrics between MLLMs were also evaluated using the bootstrap estimates. A Bonferroni correction was applied, resulting in a two-tailed threshold of statistical significance set at 0.00033. The statistical analysis was conducted using the R programming language for statistical computation, Version 4.4.0 (R Core Team (2024), R Foundation for Statistical Computing, Vienna, Austria).

## Results

### Cohort summary

A total of 136 eyes from 76 patients were included, after excluding 21 eyes due to our exclusion criteria (other retina pathology, recent intraocular surgery, history of retina surgery), and 7 due to poor image quality. The causes for exclusion of images are summarized in Supplemental Table 2. Average patient age was 81.1 (95% CI: 79.1, 83.2) years, with 69.7% being female (Table 1). There were no significant differences between subjects with AMD and without AMD in regard to age, race, ethnicity, or sex. For disease status, 17 (12.5%) eyes had ‘No AMD’, 18 (13.2%) had ‘Early AMD’, 50 (36.8%) had ‘Intermediate AMD’, and 51 (37.5%) had ‘Advanced AMD’ (Table 1). To compare human retinal specialist graders as we established a ground truth, interobserver agreement was evaluated with a weighted kappa analysis (this used squared distances to account for the relative distance between ordinal grades). Agreement was found to be 0.805 (*P* < 0.001), indicating a high level of agreement. Eighteen (13.2%) eyes had initial grader disagreement requiring adjudication by a senior retina specialist to establish final grades. Fourteen of these represented disagreements between different AMD severity levels and 4 represented disagreements between AMD and non-AMD status. Table 2 summarizes the performance of each in terms of accuracy, sensitivity, specificity, precision, F1, and agreement/Cohen’s kappa.

**Table 1:** Cohort Demographic and Ground Truth Diagnosis Summary. Cohort demographic characteristics are presented. Number of eyes and percentage of the cohort are also displayed per diagnosis, as determined by human grader consensus. Means are presented with 95% confidence intervals. AMD = age-related macular degeneration.

**Cohort Demographics** *(n* = *76 subjects)*	
**Age**	81.1 (79.1, 83.2)
**Race**	
Asian	6 (7.9%)
Black or African American	2 (2.6%)
Other Race or Mixed Race	5 (6.6%)
Unreported	2 (2.6%)
White	61 (80.3%)
**Ethnicity**	
Not Hispanic, Latino(a), or Spanish origin	67 (88.2%)
Other Hispanic, Latino(a) or Spanish origin	2 (2.6%)
Unreported	7 (9.2%)
**Sex**	
Female	53 (69.7%)
Male	23 (30.3%)
**Cohort Ground Truth Diagnosis** *(n* = *136 eyes)*	
**Disease Status** AMD No AMD	119 (87.5%) 17 (12.5%)
**Disease Severity** No AMD Early AMD Intermediate AMD Advanced AMD	17 (12.5%) 18 (13.2%) 50 (36.8%) 51 (37.5%)

**Table 2:** MLLM Accuracy, Sensitivity, Specificity, Precision, F1, and Agreement for All Prompts. Performance of MLLM classification of images for each prompt. Performance metrics are presented with 95% confidence intervals.

**Prompt 1** (Binary disease classification)						
MLLM	Accuracy	Sensitivity	Specificity	Precision	F1	Cohen’s kappa (Unweighted)
ChatGPT-4o	0.824 (0.743, 0.875)^**1**^	0.832 (0.760, 0.893)^**1**^	0.765 (0.529, 0.938)	0.961 (0.916, 0.990)	0.892 (0.848, 0.931)^**1**^	0.425 (0.233, 0.599)^**1**^
Claude 3.5 Sonnet	0.301 (0.221, 0.375)^**4,5**^	0.227 (0.156, 0.304)^**4,5**^	0.824 (0.615, 1.000)	0.900 (0.756, 1.000)	0.362 (0.264, 0.461)^**4,5**^	0.016 (−0.050, 0.074)^**5**^
Gemini 1.5 Pro	0.669 (0.581, 0.743)	0.647 (0.560, 0.727)	0.824 (0.589, 0.957)	0.963 (0.912, 1.000)	0.774 (0.702, 0.833)	0.237 (0.115, 0.381)
Perplexity Sonar Large	0.816 (0.735, 0.868)	0.815 (0.744, 0.879)	0.824 (0.600, 1.000)	0.970 (0.928, 1.000)	0.886 (0.837, 0.925)	0.432 (0.251, 0.606)
**Prompt 2** (Severity classification**)**						
MLLM	Accuracy	Sensitivity	Specificity	Precision	F1	Cohen’s kappa (Unweighted)
ChatGPT-4o	0.426 (0.338, 0.500)	0.299 (0.265, 0.347)	0.773 (0.757, 0.792)	0.731 (0.567, 0.822)	0.424 (0.360, 0.486)	0.095 (0.031, 0.176)
Claude 3.5 Sonnet	0.419 (0.331, 0.500)	0.440 (0.353, 0.531)	0.791 (0.764, 0.817)	0.603 (0.507, 0.686)	0.509 (0.427, 0.590)	0.178 (0.077, 0.281)
Gemini 1.5 Pro	0.426 (0.338, 0.500)	0.337 (0.283, 0.406)	0.775 (0.762, 0.794)	0.683 (0.558, 0.856)	0.452 (0.382, 0.535)	0.110 (0.051, 0.195)
Perplexity Sonar Large	0.463 (0.368, 0.537)	0.323 (0.283, 0.373)	0.789 (0.769, 0.812)	0.559 (0.379, 0.788)	0.409 (0.327, 0.487)	0.157 (0.075, 0.255)
**Prompt 3** (Open-ended diagnosis)						
MLLM	Accuracy	Sensitivity	Specificity	Precision	F1	Cohen’s kappa (Unweighted)
ChatGPT-4o	0.478 (0.390, 0.559)^**1,2,3**^	0.403 (0.316, 0.492)^**1,2,3**^	1.000 (1.000, 1.000)	1.000 (1.000, 1.000)	0.575 (0.477, 0.656)^**1,2,3**^	0.145 (0.083, 0.226)^**1,2,3**^
Claude 3.5 Sonnet	0.184 (0.118, 0.250)	0.067 (0.026, 0.116)	1.000 (1.000, 1.000)	1.000 (1.000, 1.000)	0.126 (0.050, 0.217)	0.018 (0.006, 0.039)
Gemini 1.5 Pro	0.154 (0.093, 0.213)	0.034 (0.008, 0.070)	1.000 (1.000, 1.000)	1.000 (1.000, 1.000)	0.065 (0.017, 0.128)	0.009 (0.002, 0.022)
Perplexity Sonar Large	0.162 (0.096, 0.221)	0.050 (0.016, 0.092)	0.941 (0.769, 1.000)	0.857 (0.400, 1.000)	0.095 (0.033, 0.174)	−0.002 (−0.048, 0.020)
**Prompt 4** (Multiple-choice diagnosis)						
MLLM	Accuracy	Sensitivity	Specificity	Precision	F1	Cohen’s kappa (Unweighted)
ChatGPT-4o	0.691 (0.603, 0.757)^**1,2**^	0.655 (0.569, 0.735)^**1,2**^	0.941 (0.779, 1.000)	0.987 (0.949, 1.000)	0.788 (0.723, 0.846)^**1,2**^	0.297 (0.178, 0.440)^**1,2**^
Claude 3.5 Sonnet	0.272 (0.191, 0.346)^**5**^	0.168 (0.106, 0.239)^**5**^	1.000 (1.000, 1.000)	1.000 (1.000, 1.000)	0.288 (0.194, 0.385)^**5**^	0.048 (0.024, 0.087)^**5**^
Gemini 1.5 Pro	0.331 (0.243, 0.404)^**6**^	0.244 (0.168, 0.325)^**6**^	0.941 (0.769, 1.000)	0.967 (0.871, 1.000)	0.389 (0.290, 0.482)^**6**^	0.057 (0.011, 0.108)
Perplexity Sonar Large	0.603 (0.507, 0.676)	0.546 (0.457, 0.629)	1.000 (1.000, 1.000)	1.000 (1.000, 1.000)	0.707 (0.629, 0.776)	0.231 (0.137, 0.341)

AMD = age-related macular degeneration; MLLM = multimodal large language model. Significant differences (*P* < 0.00033, per Bonferroni correction) between models for overall accuracy, sensitivity, and specificity are denoted by the following: ^**1**^ChatGPT vs. Claude; ^**2**^ChatGPT vs. Gemini; ^**3**^ChatGPT vs. Perplexity; ^**4**^Claude vs. Gemini; ^**5**^Claude vs. Perplexity; ^**6**^Gemini vs. Perplexity.

### AMD vs. No AMD classification (prompt 1)

**Fig. 3:**
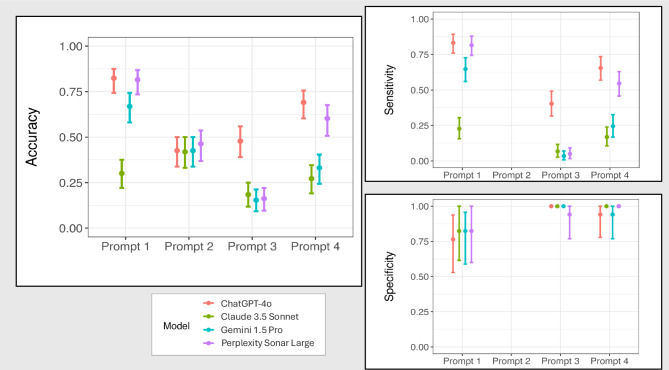
MLLM Accuracy, Sensitivity, and Specificity Comparisons. For individual prompts, significant differences (*P* < 0.00033) between models are present where error bars do not overlap.

Prompt 1 requested the four MLLMs to grade images as more likely having or not having AMD. Accuracy was highest for ChatGPT-4o [0.824 (95% CI: 0.743, 0.875)], followed by Perplexity Sonar Large [0.816 (95% CI: 0.735, 0.868)], Gemini 1.5 Pro [0.669 (95% CI: 0.581, 0.743)], and Claude 3.5 Sonnet [0.301 (95% CI: 0.221, 0.375)]. Significant differences were present between the lowest performing (Claude 3.5 Sonnet) and the other three MLLMs (*P* < 0.00033) (Fig. 3). The same significant differences were found with F1 score, which was led by ChatGPT-4o [0.892 (0.848, 0.931)], Perplexity Sonar Large [0.886 (95% CI: 0.837, 0.925)], and Gemini 1.5 Pro [0.774 (95% CI: 0.702, 0.833)], followed by Claude [0.362 (95% CI: 0.264, 0.461)]. Sensitivity was highest for ChatGPT-4o [0.832 (95% CI: 0.760, 0.893)], followed by Perplexity Sonar Large [0.815 (95% CI: 0.744, 0.879)], Gemini 1.5 Pro [0.647 (95% CI: 0.560, 0.727)], and Claude 3.5 Sonnet [0.227 (95% CI: 0.156, 0.304)], with significant differences again only between Claude 3.5 Sonnet and the other models (*P* < 0.00033). Specificity was non-significantly different between Claude 3.5 Sonnet [0.824 (95% CI: 0.615, 1.000)], Gemini 1.5 Pro [0.824 (95% CI: 0.589, 0.957)] and Perplexity Sonar Large [0.824 (95% CI: 0.600, 1.000)], followed by ChatGPT-4o [0.765 (95% CI: 0.529, 0.938)]. There were no significant differences in specificity between models.

### AMD severity classification (prompt 2)

Prompt 2 requested the four MLLMs to grade images for the severity of AMD (ranging from ‘No AMD’ to ‘Advanced AMD’) based on the AREDS grading criteria. In all comparisons between models, there were no significant differences. Accuracy was highest for Perplexity Sonar Large [0.463 (95% CI: 0.368, 0.537)], followed by ChatGPT-4o [0.426 (95% CI: 0.338, 0.500)], Gemini 1.5 Pro [0.426 (95% CI: 0.338, 0.500)], and Claude 3.5 Sonnet [0.419 (95% CI: 0.331, 0.500)]. Cohen’s kappa coefficients demonstrated universally poor agreement with human graders when calculated unweighted (range: 0.095 to 0.178), and also when calculated with squared distance weighting to account for relative nearness of responses to each other (range: −0.009 to 0.208). F1 score found no significant differences between models, and was generally poor, ranging from 0.409 (95% CI: 0.327, 0.487) to 0.509 (95% CI: 0.427, 0.590) overall.

### Open-ended diagnosis (prompt 3)

Prompt 3 requested the four MLLMs to give the most likely diagnosis, if any, based on the image. This was intended to test MLLM performance in a query unspecific to AMD, and to establish a baseline accuracy for a broader diagnostic use case. Accuracy was highest for ChatGPT-4o [0.478 (95% CI: 0.390, 0.559)], followed by Claude 3.5 Sonnet [0.184 (95% CI: 0.118, 0.250)], Perplexity Sonar Large [0.162 (95% CI: 0.096, 0.221)], and Gemini 1.5 Pro [0.154 (95% CI: 0.093, 0.213)]. Significant differences were present between ChatGPT-4o and the other MLLMs (*P* < 0.00033). Sensitivity was significantly higher (*P* < 0.00033) for ChatGPT-4o [0.403 (95% CI: 0.316, 0.492)], followed by Claude 3.5 Sonnet [0.067 (95% CI: 0.026, 0.116)], Perplexity Sonar Large [0.050 (95% CI: 0.016, 0.092)] and Gemini 1.5 Pro [0.034 (95% CI: 0.008, 0.070)]. Specificity was roughly equal between models, at [1.000 (95% CI: 1.000, 1.000)] for ChatGPT-4o, Claude 3.5 Sonnet and Gemini 1.5 Pro, followed by Perplexity Sonar Large [0.941, (95% CI: 0.769, 1.000)], with no significant differences between models. F1 score was also significantly highest for ChatGPT-4o at 0.575 (95% CI: 0.477, 0.656), followed by the others [range: 0.065 (95% CI: 0.017, 0.128)—0.126 (95% CI: 0.050, 0.217)].

### Multiple-choice diagnosis (prompt 4)

Prompt 4 requested the four MLLMs to give the most likely diagnosis, if any, based on the image from a multiple-choice list of 12 common retina conditions (age-related macular degeneration, central serous chorioretinopathy, diabetic retinopathy, epiretinal membrane, glaucoma, hypertensive retinopathy, macular edema, no apparent retinopathy, retinal detachment, retinal vascular occlusion, retinitis pigmentosa, or other pathology). This was again intended to test MLLM performance in a query unspecific to AMD, and to establish a baseline accuracy for a broader diagnostic use case. Accuracy was highest for ChatGPT-4o [0.691 (95% CI: 0.603, 0.757)], followed by Perplexity Sonar Large [0.603 (95% CI: 0.507, 0.676)], Gemini 1.5 Pro [0.331 (95% CI: 0.243, 0.404)], and Claude 3.5 Sonnet [0.272 (95% CI: 0.191, 0.346)]. Significant differences (*P* < 0.00033) were present between the top two (ChatGPT-4o, Perplexity Sonar Large) and the bottom two (Claude 3.5 Sonnet, Gemini 1.5 Pro) MLLMs. F1 scores found the same significant differences, with ChatGPT-4o [0.788 (95% CI: 0.723, 0.846)] and Perplexity Sonar Large [0.707 (95%CI: 0.629, 0.776)] leading the others. Sensitivity was highest for ChatGPT-4o [0.655 (95% CI: 0.569, 0.735)], followed by Perplexity Sonar Large [0.546 (95% CI: 0.457, 0.629)], Gemini 1.5 Pro [0.244 (95% CI: 0.168, 0.325)] and Claude 3.5 Sonnet [0.168 (95% CI: 0.106, 0.239)], with significant differences (*P* < 0.00033) again present between the top two and bottom two models. Specificity was similar for all models, at [1.000 (95% CI: 1.000, 1.000)] for Claude 3.5 Sonnet and Perplexity Sonar Large, followed by [0.941 (95% CI: 0.779, 1.000)] for ChatGPT-4o and Gemini 1.5 Pro [0.941 (95% CI: 0.769, 1.000)], and with no significant differences between models.

### Error analysis

Additional analyses looked at misclassifications and accuracy results stratified by ground truth diagnosis (Supplemental Table 3) to determine any patterns in MLLM misclassification of disease. Sample MLLM outputs are provided for reference in Supplemental Fig. 2. Overall, accuracy varied across prompts and models. ‘Early AMD’ was detected with poor accuracy on average across all prompts (mean 19.8% all-model average), and ‘No AMD’ was more accurately identified (mean 72.8% all-model average). For severity classification (Prompt 2), ‘No AMD’ was poorly identified (mean 14.7% all-model average). ‘Intermediate AMD’ and ‘Advanced AMD’ were identified with high variation between prompts and models.

An inter-model agreement analysis was also performed alongside confusion matrices (Supplemental Table 4). For Prompt 1, agreement was highest between the two top performing models, ChatGPT-4o and Perplexity Sonar Large (80.1%), with other models showing variable agreement (39.0% to 64.0%). For Prompt 2, models exhibited a shared bias toward overclassifying ‘Intermediate AMD’, which contributed to elevated inter-model agreement. Prompt 3 showed high overall pairwise inter-model agreement (81.6%), driven by frequent overclassification of non-AMD pathologies for this open-ended diagnosis prompt. Prompt 4 demonstrated moderate overall pairwise agreement (65.1%), with the highest agreement again between ChatGPT-4o and Perplexity Sonar Large (79.4%). Models again heavily favored non-AMD classifications in this multiple-choice prompt.

## Discussion

The present study evaluated and compared the capabilities of four popular MLLMs in detecting AMD and grading the severity of disease from UWF fundus images. While other deep learning algorithms have been deployed in similar contexts in this and other ophthalmic diseases, the accuracy of various MLLMs for this application remains unclear. We found that results varied substantially between MLLMs, emphasizing that models cannot be interchanged with the expectation of similar results. Consistent with prompt engineering concepts^13^, question type and framing also strongly influenced outcomes. Overall, MLLMs showed some promise in assessing images for AMD, particularly in binary disease classification. However, the levels of performance we observed suggest that these technologies are not yet ready for safe clinical implementation.

The best performance was seen in binary disease classification (Prompt 1), with accuracies and F1 scores exceeding 80% for ChatGPT-4o and Perplexity Sonar Large. These results (ChatGPT-4o sensitivity/specificity: 0.832/0.765; Perplexity Sonar Large sensitivity/specificity: 0.815/0.824) are slightly below, but comparable to, those achieved by optometrists (84.5% sensitive, 88.0% specific) classifying fundus images for the presence or absence of AMD^14^. Notably, optometrists play an essential role in screening for asymptomatic AMD^15^.

Compared to physician accuracy in grading AMD severity (75.8%) with a 4-class AREDS scale^16^ (Prompt 2), MLLMs fell short, failing to attain greater than 46.3% accuracy, and only poor F1 scores (range: 0.409–0.509). Together with the results of Prompt 1, this suggests MLLMs may be nearer to clinical readiness for use cases like AMD screening, rather than for precision diagnosis and monitoring of disease severity. It is worth noting that relatively low sample sizes for the ‘Early AMD’ and ‘No AMD’ groups may have limited the power of this comparison.

Prompts 3 and 4 evaluated the capabilities of MLLMs as nonspecific diagnostic tools asked to make a diagnosis based on fundus imaging alone. With accuracies ranging from 0.154 to 0.691, reliability was both inconsistent and likely insufficient for this clinical use case. ChatGPT-4o’s ability, However, to accurately diagnose AMD 69.1% of the time from a fundus image alone [F1 score of 0.788 (0.723, 0.846)], without prompting specific to AMD, is promising at this relatively early stage in the technology’s development.

In comparing models, ChatGPT-4o demonstrated significantly better performance Than its counterparts in 3 of 4 prompts, with Perplexity Sonar Large outperforming Claude 3.5 Sonnet and Gemini 1.5 Pro in 2 of 4. This suggests that at least the tested models of Claude and Gemini may lag in accuracy. However, as this study demonstrated, results can vary widely between prompts, and further experimentation with additional prompts and in other contexts would help to identify a clear MLLM leader in the space of ophthalmic imaging analysis.

Differences in model architectures or training data could be responsible for variations in performance. However, it is difficult to determine the specific driving factors behind our results for several reasons. First, the performance of MLLMs varies notably depending on the prompt and the specific medical imaging task, as demonstrated in this study and other previous literature. For example, ChatGPT-4o outperformed Gemini 1.5 Pro in the present study, as well as in a separate study focused on diagnosing corneal disease where ChatGPT-4o achieved the highest accuracy, followed by Claude 3.5 Sonnet and then Gemini 1.5 Flash^17^. However, the opposite was observed in a recent investigation where MLLMs interpreted magnetic resonance brain imaging^18^, and another study evaluating MLLM responses to ocular inflammation questions using patient imaging found no significant performance difference between ChatGPT-4o and Gemini 1.5 Pro^19^. Secondly, there is currently a lack of publicly available data regarding many MLLM technical specifications and training data, as companies such as OpenAI and Google move away from open-source models and public disclosure around training data^17,20^. This makes fully informed comparisons difficult. However, we could speculate that the superior performances of ChatGPT-4o and Perplexity Sonar Large observed in this study could be due to the use of training data better suited to this particular context, or perhaps certain architectural differences. ChatGPT-4o has been noted to use particularly vast and diverse datasets^17^, and Perplexity models employ retrieval-augmented generation (RAG) with live information retrieval^21^, which may enhance the LLM’s accuracy when new data becomes available online after the training cutoffs of other models. Gemini 1.5 Pro is a mid-sized model optimized for flexible scaling across a wide range of contexts^17,18^, which could limit performance in our very specific medical task of interest. Several studies have also found Gemini to underperform in visual analysis tasks, highlighting a possible relative weakness in interpreting complex medical imagery^17,22^. Claude 3.5 Sonnet incorporates post-training refinement through human feedback, where reviewers help align model outputs to prioritize quality and safety, often resulting in more conservative results^17^. This is consistent with our results, where Claude 3.5 Sonnet was found to be more specific at the expense of low sensitivity. This was also the case with Gemini 1.5 Pro, suggesting these models may have favored more conservative approaches overall. Identifying the specific technical factors driving performance differences we observed is challenging. However, further comparisons across diverse ophthalmic imaging settings may help to clarify any broader trends.

Error analysis revealed accuracy differences across ground truth AMD severities. Average accuracy was higher for ‘No AMD’ eyes and lowest for ‘Early AMD’ eyes, with results for intermediate and advanced cases falling somewhere between. This could suggest that MLLMs recognize healthy eyes more reliably, and perhaps are more sensitive to certain high-risk features such as neovascularization and geographic atrophy found in advanced stages. Poor recognition of ‘Early AMD’ eyes, particularly in Prompts 3 and 4, was reasonably expected, as this least severe form of AMD has the least obvious pathological features. Inter-model agreement analysis with confusion matrices was performed to evaluate whether inherent case difficulty might be contributing to misclassifications, or if errors were more driven by model limitations. The high agreement between top-performing models (ChatGPT-4o and Perplexity Sonar Large, 80.1%) in Prompt 1 suggested that lower-performing models may have suffered from limitations handling the task, rather Than inherent case difficulty. In Prompt 2, we observed shared biases across MLLMs with overclassification of ‘Intermediate AMD’. A pattern bias such as this might suggest imbalances in training data, as deep learning models can become biased toward the majority of data^23^. While it is difficult to determine what may constitute the majority in the training data, this would be consistent with the relatively higher prevalence of early (including ‘Intermediate AMD’) vs. late-stage AMD in the US population^24^. Overclassifications of non-AMD pathologies were observed in Prompt 3, with relatively high agreement between models. This was likely related to higher task difficulty, but importantly highlights reduced MLLM performance when prompts did not drive at analysis for a particular pathology of interest, as Prompts 1 and 2 did. Prompt 4 again demonstrated relatively high agreement between the top performing models (ChatGPT-4o and Perplexity Sonar Large, 79.4%), as well as overclassification of non-AMD eyes and agreement between MLLMs in these negative responses. Model limitations may have hindered lower performing models, which did relatively worse with this higher difficulty task. Overall, it is difficult to determine the precise reasons behind error trends in a single study and use case. Additional studies focusing on larger MLLM error trends in ophthalmic imaging may help to confirm these hypotheses and uncover additional driving forces.

Taken together, results from this study suggest these MLLMs are not currently ready for clinical use in AMD detection. However, results for binary disease detection may approach those of human graders. Furthermore, these are early models that are expected to improve quickly with further training and have already done so recently^25^. Research using other deep-learning AI algorithms has shown performance that is on par with human graders for grading disease severity^26^, and as high as 98% accuracy in detecting referrable (intermediate to advanced) vs. non-referrable (none to early) AMD from color fundus photos^1^. This demonstrates the potentially high performance achievable by an AI screening tool for automated detection. However, these tools are often limited as disease-specific algorithms, trained on data specific to AMD to recognize a single disease^1^. This likely increases accuracy, but also narrows the scope of possible applications for the tool.

MLLMs have the distinct advantage of being generalized tools trained on a much broader set of data^27^. They are able to respond to myriad unique queries, and therefore may be able to simultaneously assist ophthalmologists through many different applications^10,27^. For example, while screening for AMD in a patient, MLLMs could also assess the patient’s fundus imaging for glaucoma^5^, as well as analyze OCT and other imaging modalities to check for numerous other pathologies^28^. Following the visit, the MLLM could generate an after-visit summary^29^ and answer patient questions regarding their diagnosis with relatively high accuracy^30^. MLLMs can also process multiple types of information (i.e. visual and textual) simultaneously, allowing for a potentially deeper and more holistic understanding of a patient’s diagnosis, prognosis, or best treatment options^27^. As another example, when analyzing a fundus image an MLLM could simultaneously consider relevant patient history from clinical notes that might influence the interpretation of ambiguous findings. Other advantages include the potential for users to converse with the technology, ask follow-up questions, ask for detailed explanations, or to use the MLLM as an educational tool while in training^25,27^. MLLMs may also be cost-effective in comparison to other deep learning tools that are expensive to train for specific use cases^27^. Compared to other algorithms, a limited amount of MLLM fine-tuning can significantly improve accuracy, as they are trained on large amounts of data at baseline.

While MLLMs will potentially be highly useful, careful consideration of the safety and ethical implications of deploying them to screen for and monitor ophthalmic disease is critical. Performance may improve with time, but even a small number of errors or hallucinations can cause substantial harm to patients^31^. Diligent auditing of MLLM outputs and provided reasoning behind answers may mitigate risks to some degree, but the “black box” nature of these and other AI tools could make complete understanding difficult. These tools may also learn from our own biases if present in training data, which should include diverse and representative datasets^27^. For example, in this study MLLMs had not necessarily been trained on unbiased datasets, but likely images available on the internet. This could reduce the applicability of results for certain populations. Further research in this area will be necessary prior to real-world implementation. Patient privacy will also need to be prioritized and protected. While this study used only anonymized data, expanded use cases of MLLMs that may include medical records or other identifying information would necessitate the establishment of clear guidelines and regulations^28^.

This study was limited by its retrospective nature with a relatively small number of cases for some disease severity levels. Images were from a single academic center, and exclusion criteria may have contributed some degree of selection bias. The intended end users (clinicians) for our use case would need to screen patient types that were excluded in this study, including those with poor image quality and complex ocular histories. However, strict exclusion criteria applied alongside anonymization were used to maintain data integrity. Observer bias was mitigated through the utilization of trained retinal specialist graders, who independently graded images, with any disagreements adjudicated by a senior retina specialist. Consensus grading using standardized AREDS criteria was applied to reduce subjective variability and increase reliability, though the influence of subjectivity, particularly in borderline cases, remains. A single camera type was used to obtain images, and only UWF imaging was provided to MLLMs to establish baseline performance using this single commonly used modality. Clinical diagnosis typically benefits from additional patient history and imaging, and imaging is often not graded using published grading criteria in the clinical setting. This is a potential source of information bias that could limit clinical generalizability of our findings to some degree. As UWF imaging is one of the most cost-effective modalities to screen for retinal disorders^6^, however, this single-modality application may help elucidate minimal requirements for accurate diagnosis and/or diagnosis in lower-resource settings. Finally, some of the MLLM models studied here have been replaced with updated versions, limiting reproducibility of the work.

Further studies around using MLLMs for AMD can widen the scope of automated disease detection to include disease prognosis and even treatment recommendations. Multimodal imaging inputs such as OCT, OCT angiography, and fluorescein angiography could also be analyzed in tandem along with demographics and history to see if MLLMs achieve higher accuracy^2,6^. Studies assessing MLLM detection of particular prognostic features, such as reticular pseudodrusen on OCT^7^, would provide further information on the diagnostic utility of MLLMs, and identify targets for fine-tuning. Additionally, given the treatment implications for wet versus dry AMD, future studies assessing the abilities of MLLMs to distinguish between nAMD and geographic atrophy could provide further insights into clinical utility. Greater accuracy may also be achievable with different prompts, few-shot learning^32^, or by training these MLLMs for the specific purpose of AMD image analysis^27^. Lastly, requesting MLLMs to include probability estimates along with answers may be useful to calculate receiver operating characteristic areas under the curve (AUC-ROC), and to stratify accuracy results by relative confidence expressed by the algorithm.

In conclusion, the present study represents a preliminary evaluation of four leading MLLMs in fundus image-based detection and grading of AMD severity. MLLMs show promise as emerging technologies that may one day accurately and efficiently evaluate images for this disease, thereby supporting screening and monitoring efforts while protecting resources. However, the results of this analysis suggest that these MLLMs could not be safely and reliably implemented in clinical settings in their present forms. As the field of ophthalmology continues to explore clinical adoption of AI technologies, rigorous and ongoing validation of these and prospective models is recommended to protect high standards of safety and patient care.

## Supplementary Information

Supplementary Information.

## Data Availability

All data regarding this report can be accessed with permission (requests can be directed to JM or SB), and we take full responsibility for the integrity of the data and its analysis.

## Abbreviations

AMD: Age-related macular degeneration
nAMD: Neovascular age-related macular degeneration
OCT: Optical coherence tomography
AI: Artificial intelligence
DL: Deep learning
LLMs: Large language models
MLLMs: Multimodal large language models
UWF: Ultrawide field
UCSD: University of California, San Diego
AREDS: Age-Related Eye Disease Study
MC: Multiple-choice
CI: Confidence interval
RAG: Retrieval-augmented generation
AUC-ROC: Receiver operating characteristic areas under the curve
