# Supplementary material for: Evaluating the clinical utility of multimodal large language models for detecting age-related macular degeneration from retinal imaging
Source: Sci Rep. 2025 Sep 26;15:33214. doi: 10.1038/s41598-025-18306-1 (PMC12475039; doi:10.1038/s41598-025-18306-1)
Supplement: Supplementary file 1 — Supplementary Information. [file 41598_2025_18306_MOESM1_ESM.pdf]

Supplementary Figures and Tables

Evaluating the Clinical Utility of Multimodal Large Language Models for Detecting Age-Related Macular Degeneration from Retinal Imaging

Jesse A Most[1,2], Gillian A Folk[2], Evan H Walker[3], Ines D Nagel[1,3], Nehal N Mehta[1,3], Elena Flester[2], Shyamanga Borooah*[1,3]

[1]Jacobs Retina Center, Shiley Eye Institute, University of California San Diego, La Jolla, CA, USA.
[2]School of Medicine, University of California San Diego, La Jolla, California, United States of America, 92037
[3]Viterbi Family Department of Ophthalmology and Shiley Eye Institute, University of California San Diego, California, United States of America

Supplemental Figure 1. Participant eye selection flow diagram.
AMD = age-related macular degeneration

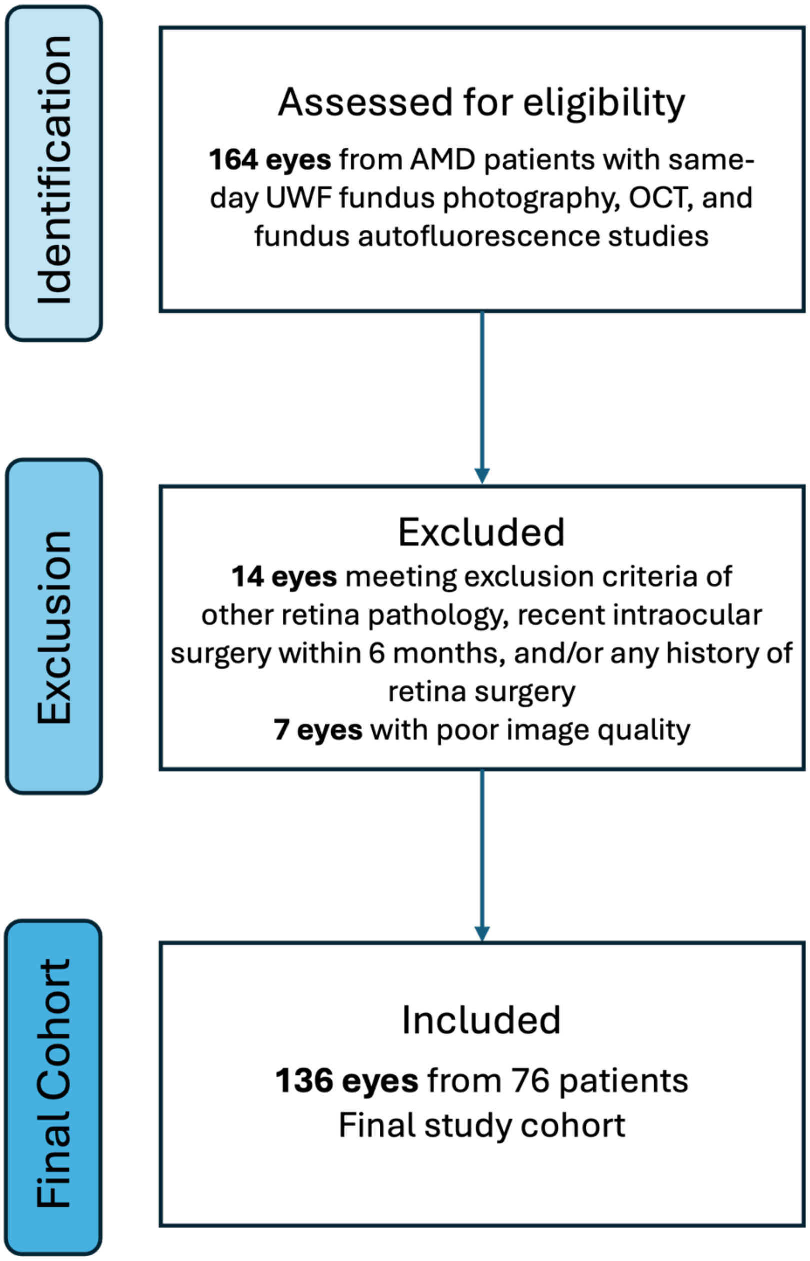

Supplemental Figure 2

Example multimodal large language model (MLLM) outputs are presented for various model and prompt combinations. Correct and incorrect response examples are shown.

Example 1
Prompt 1 (AMD presence/absence), Model: ChatGPT-4o, Correct Response
Diagnosis: AMD

Example Multimodal Large Language Model Output

Model: ChatGPT-4o, Prompt: 1 (Grading AMD presence/absence)

"Based on the image provided, the presence of numerous yellowish deposits (drusen-like features) in the macular region suggests that **age-related macular degeneration (AMD)** is a likely diagnosis. These deposits are characteristic of AMD, particularly in its early stages (dry AMD).

Correct Answer:

a) has age-related macular degeneration

Explanation:

Drusen are extracellular deposits located between the retinal pigment epithelium (RPE) and Bruch's membrane, and their presence is a hallmark of AMD. While further clinical evaluation (e.g., patient history, visual acuity testing, and optical coherence tomography) would be required for confirmation, the image strongly indicates AMD over other conditions."

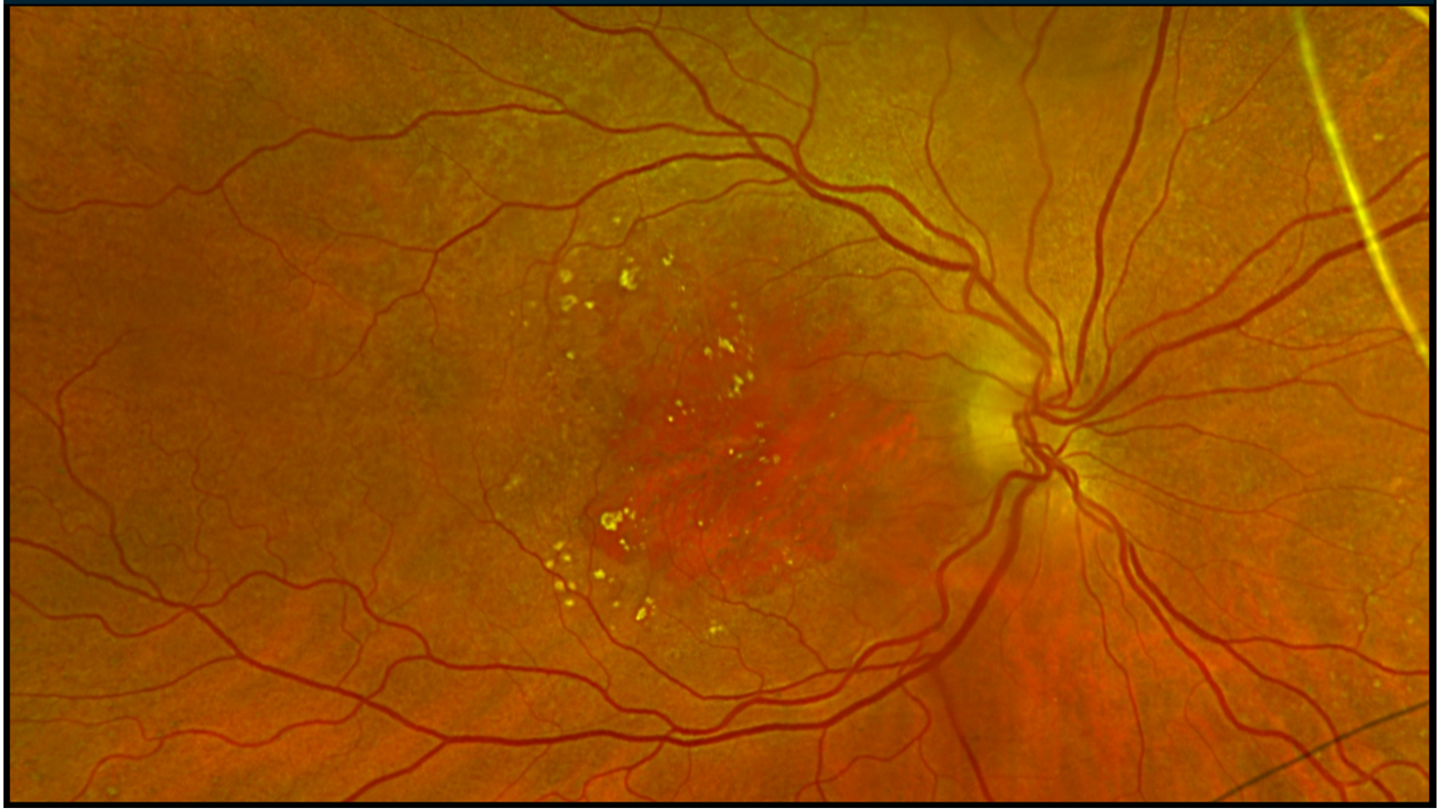

Example 2
Prompt 1 (AMD presence/absence), Model: ChatGPT-4o, Incorrect Response
Diagnosis: No AMD

Example Multimodal Large Language Model Output

Model: ChatGPT-4o, Prompt: 1 (Grading AMD presence/absence)

“Based on the image provided, the retina appears to show signs of age-related macular degeneration (AMD), including drusen (yellow deposits beneath the retina). There do not appear to be any pigmentary changes or geographic atrophy, which are also hallmark features of AMD.

Correct Answer:

a) has age-related macular degeneration

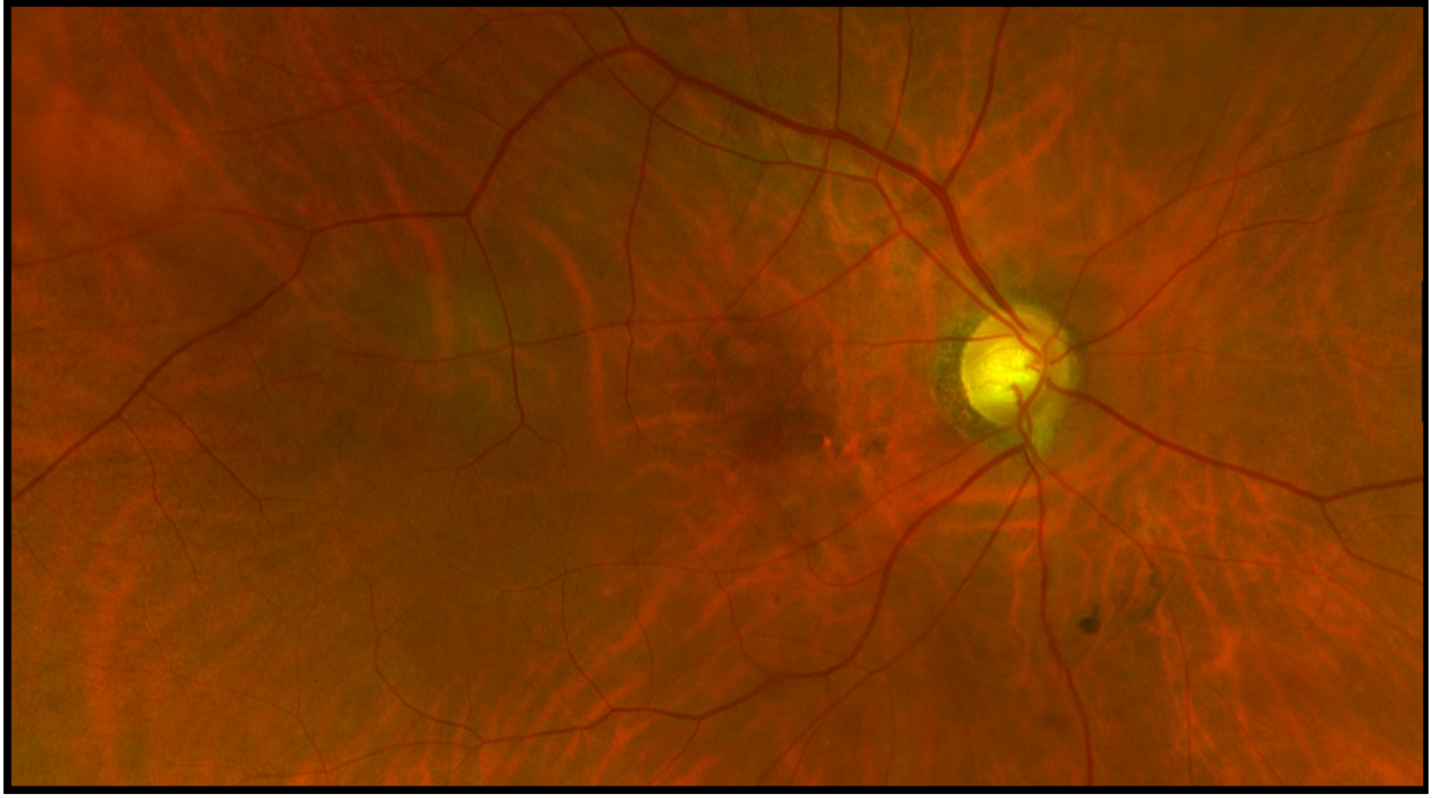

Example 3

Prompt 2 (Grading AMD severity), Model: ChatGPT-4o, Correct Response

Diagnosis: Intermediate AMD

Example Multimodal Large Language Model Output

Model: ChatGPT-4o, Prompt: 2 (Grading AMD severity)

"Based on the retinal image shown within the black grid, and applying the AREDS classification criteria, I can see evidence of multiple drusen of varying sizes. The image shows the characteristic orange-red background of a fundus photograph with the optic disc visible on the right side and a measurement grid overlay on the left.

Looking specifically at the area within the black grid and comparing to the AREDS criteria:
- There appear to be numerous intermediate drusen
- There is evidence of at least one large druse (≥125 µm in diameter) as indicated by the measurement markers on the right side of the image
- I don't see clear evidence of geographic atrophy involving the foveal center
- I don't observe definitive signs of neovascular maculopathy

Based solely on these observations within the grid area:

Intermediate AMD

This classification is most appropriate because the image demonstrates numerous intermediate drusen and at least one large druse, which are defining characteristics of AREDS category 3 (Intermediate AMD)."

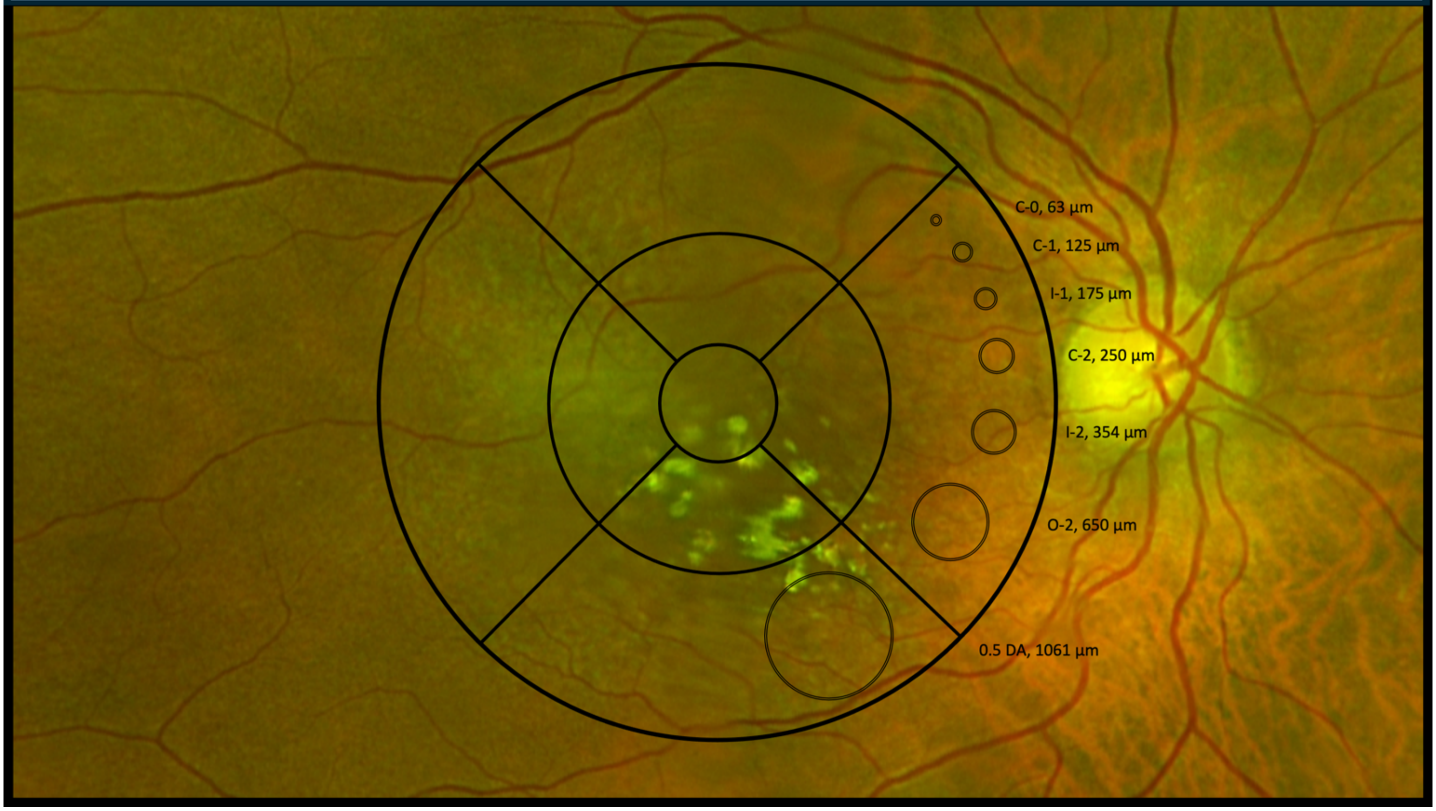

Example 4
Prompt 2 (Grading AMD severity), Claude 3.5 Sonnet, Correct Response
Diagnosis: Intermediate AMD

Example Multimodal Large Language Model Output

Model: Claude 3.5 Sonnet, Prompt: 2 (Grading AMD severity)

"Based on the retinal image provided, as a hypothetical ophthalmologist examining this fundus photograph, I would determine that it is most likely that this patient has:

Intermediate age-related macular degeneration

The image shows several key features consistent with age-related macular degeneration (AMD):

1. The yellowish deposits visible in the macular region (central area) appear to be drusen, which are hallmark features of AMD
2. There appears to be some pigmentary changes in the macula
3. The image shows measurement markers for different regions (C-0, C-1, I-1, etc.) which are typically used in retinal imaging to document and measure macular pathology
4. The overall appearance of the macula shows alterations consistent with early to intermediate AMD

The concentric measurement circles centered on the macula (ranging from 63μm to 1061μm) are likely being used to quantify and track the extent of macular changes. Without additional information about the patient's symptoms or other diagnostic tests, this fundus image strongly suggests the presence of age-related macular degeneration rather than a normal macula."

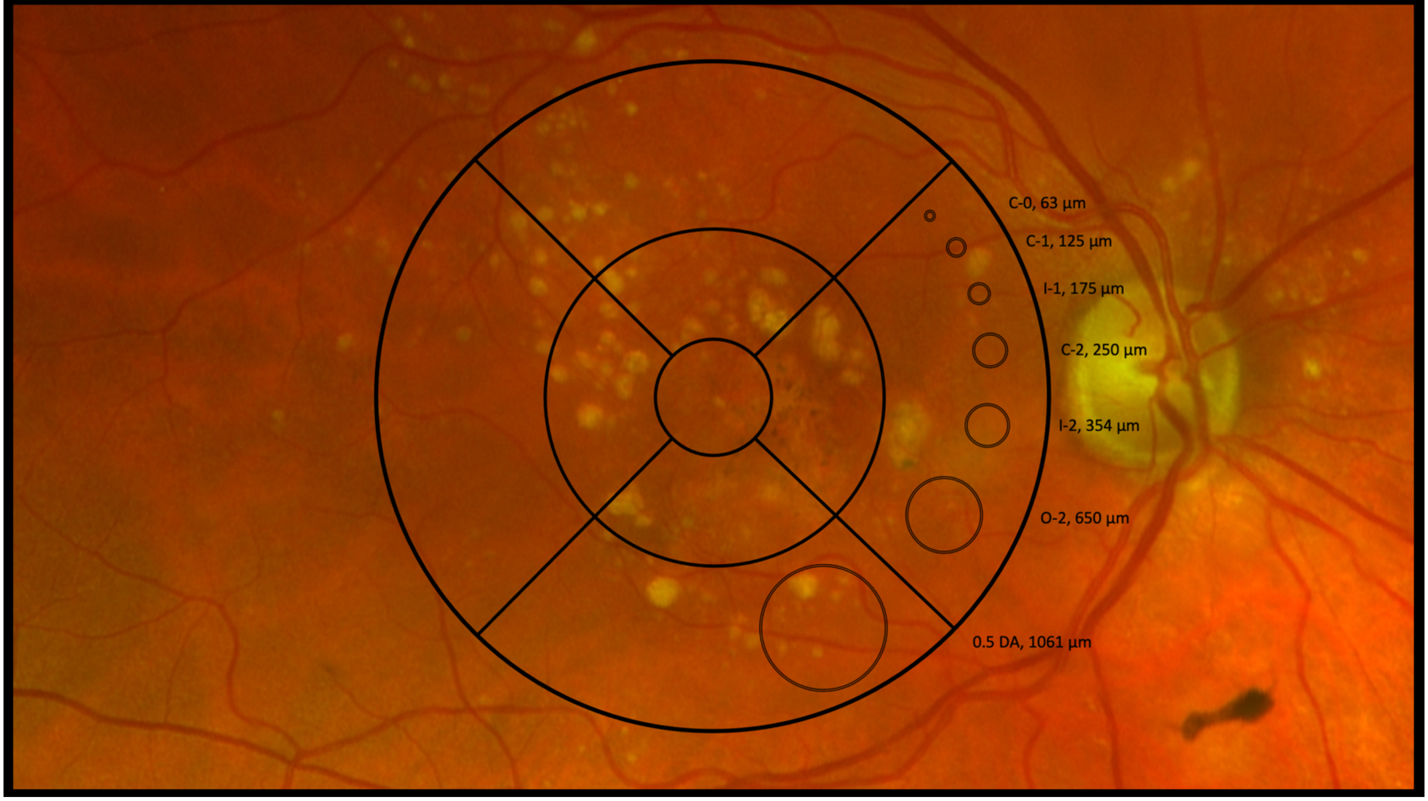

Example 5
Prompt 3 (Open-ended diagnosis), Claude 3.5 Sonnet, Incorrect Response
Diagnosis: Intermediate AMD

Example Multimodal Large Language Model Output

Model: Claude 3.5 Sonnet, Prompt: 3 (Open-ended diagnosis)

"Based on the retinal image shown, the most visible diagnosis appears to be diabetic retinopathy.

The image shows multiple yellowish-green spots and clusters scattered throughout the retina, which are consistent with diabetic retinopathy lesions. These appear to be hard exudates and microaneurysms characteristic of this condition.

The presence of numerous small, bright lesions distributed across the macula region, along with visible retinal vessels and the characteristic coloration pattern, strongly suggests diabetic retinopathy as the most evident diagnosis in this fundus photograph."

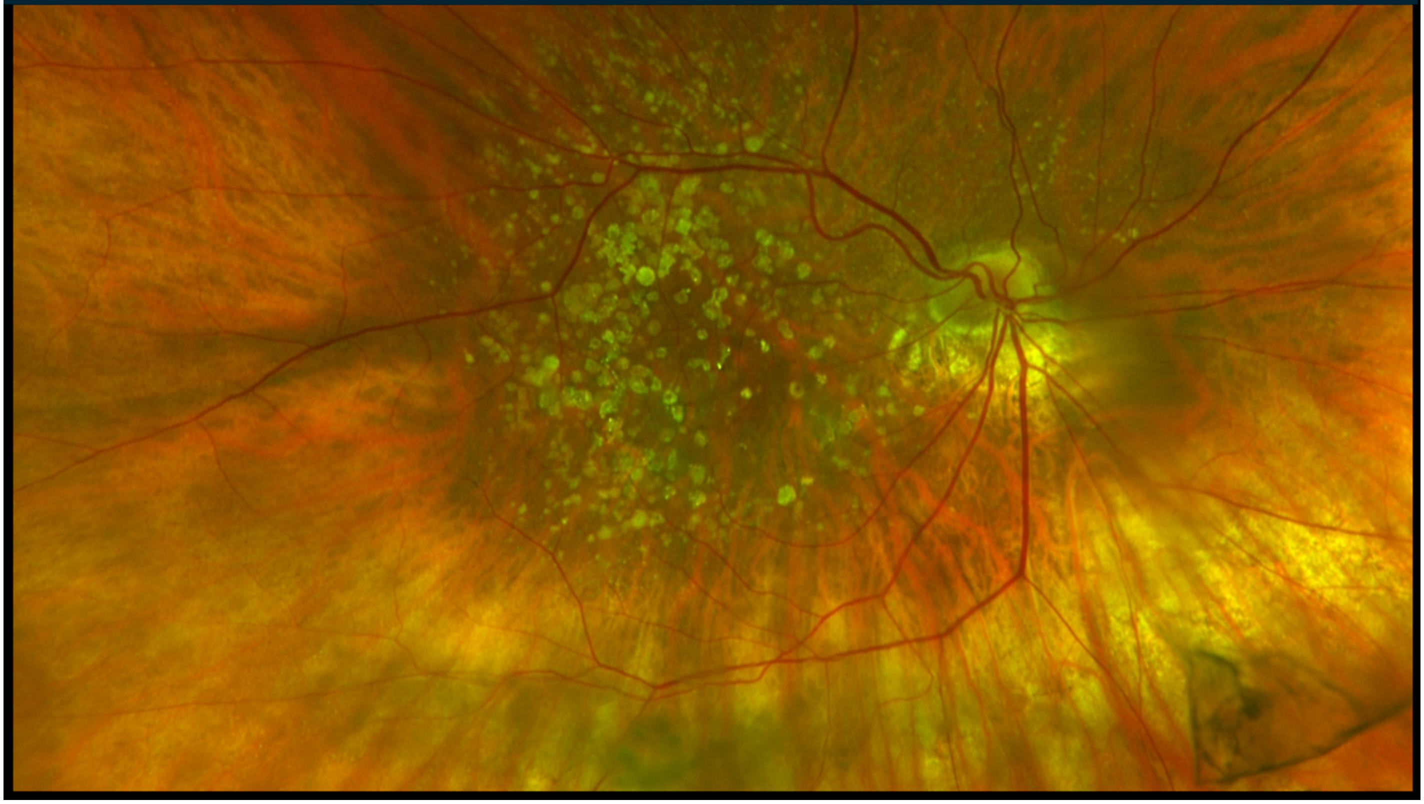

Example 6
Prompt 2 (Grading AMD severity), Gemini 1.5 Pro, Correct Response
Diagnosis: Intermediate AMD

Example Multimodal Large Language Model Output

Model: Gemini 1.5 Pro, Prompt: 2 (Grading AMD severity)

"Intermediate AMD

Explanation:

Based on the provided image and AREDS classification, the retina within the black grid shows characteristics consistent with Intermediate AMD.

Here's why:

Presence of Intermediate Drusen: The image shows multiple drusen with sizes seemingly falling within the intermediate range (63-124 µm).
Possible Large Druse: There appears to be at least one druse that could be ≥125 µm, qualifying as a large druse.
While there doesn't appear to be geographic atrophy involving the foveal center or neovascular maculopathy, the presence of numerous intermediate drusen or a single large druse is sufficient to classify this as Intermediate AMD."

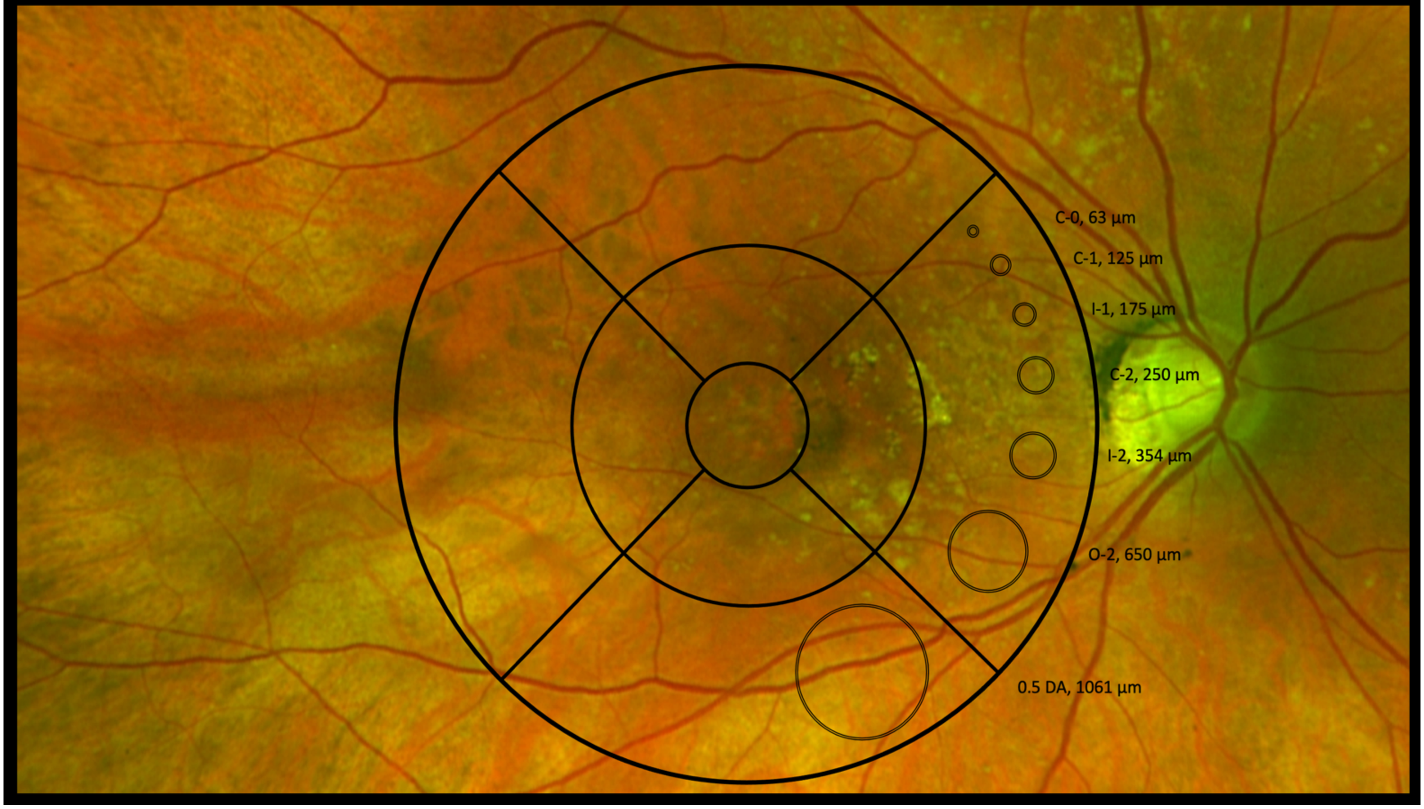

Example 7
Prompt 4 (multiple-choice diagnosis), Gemini 1.5 Pro, Incorrect Response
Diagnosis: AMD

Example Multimodal Large Language Model Output

Model: Gemini 1.5 Pro, Prompt: 4 (Multiple-choice diagnosis)

"Based on the image alone, the most likely diagnosis is **No apparent retinopathy**.

Here's why:
* **The image shows a clear view of the optic disc and retinal vessels.** There are no obvious signs of hemorrhages, exudates, neovascularization, or significant changes to the optic disc that would suggest diabetic retinopathy, hypertensive retinopathy, or retinal vascular occlusion.
* **The macula appears relatively intact.** While there is a slight yellow hue, it doesn't show the characteristic drusen or geographic atrophy seen in age-related macular degeneration, nor the fluid accumulation of macular edema.
* **There is no visible retinal detachment.** The retina appears attached throughout the visible field.
* **The optic disc appears healthy.** There is no cupping or pallor suggestive of glaucoma.
* **The retinal vessels are not attenuated or bone spicule-shaped**, which would be seen in retinitis pigmentosa.
* **There's no obvious epiretinal membrane.**
* **There is no evidence of subretinal fluid that would be expected in central serous chorioretinopathy.**
Important Note: This is based on a single image and limited information. A comprehensive eye exam and further testing would be necessary for a definitive diagnosis. The slight yellow hue in the image could potentially indicate a subtle underlying issue that would require further investigation. However, based solely on the visible features in this image, "no apparent retinopathy" is the most accurate assessment."

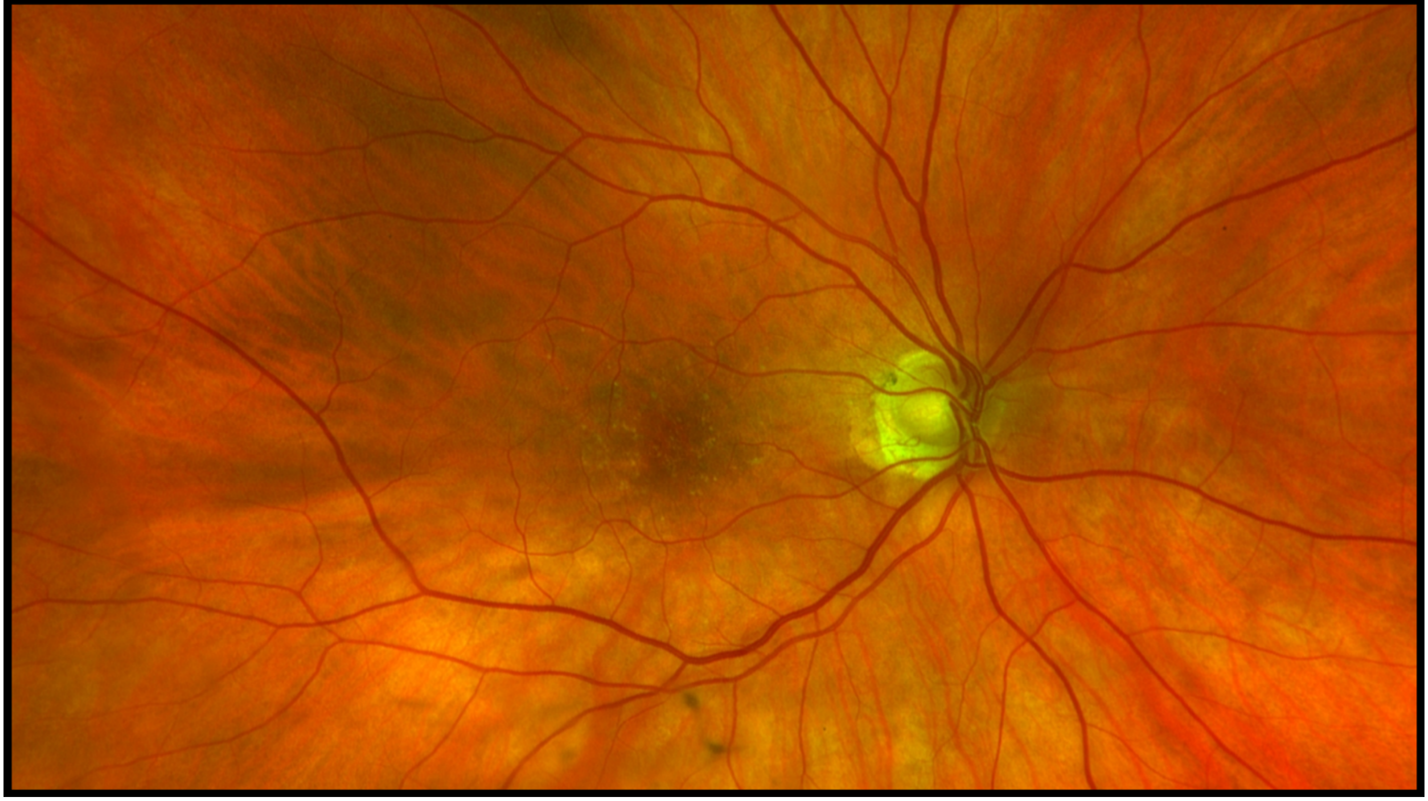

Example 8
Prompt 1 (AMD presence/absence), Perplexity Sonar Large, Correct Response
Diagnosis: AMD

Example Multimodal Large Language Model Output

Model: Perplexity Sonar Large, Prompt: 1 (AMD presence/absence)

"Answer: a) has age-related macular degeneration

Explanation:

The image shows hallmark signs consistent with age-related macular degeneration (AMD), including:

- Presence of drusen (yellowish deposits) near the macula area.
- Irregular pigmentation and areas of retinal atrophy.
- Macular changes indicating degenerative retinal tissue.

These features are characteristic indicators of AMD rather than a normal or other retinal condition, making it more likely that the patient has age-related macular degeneration."

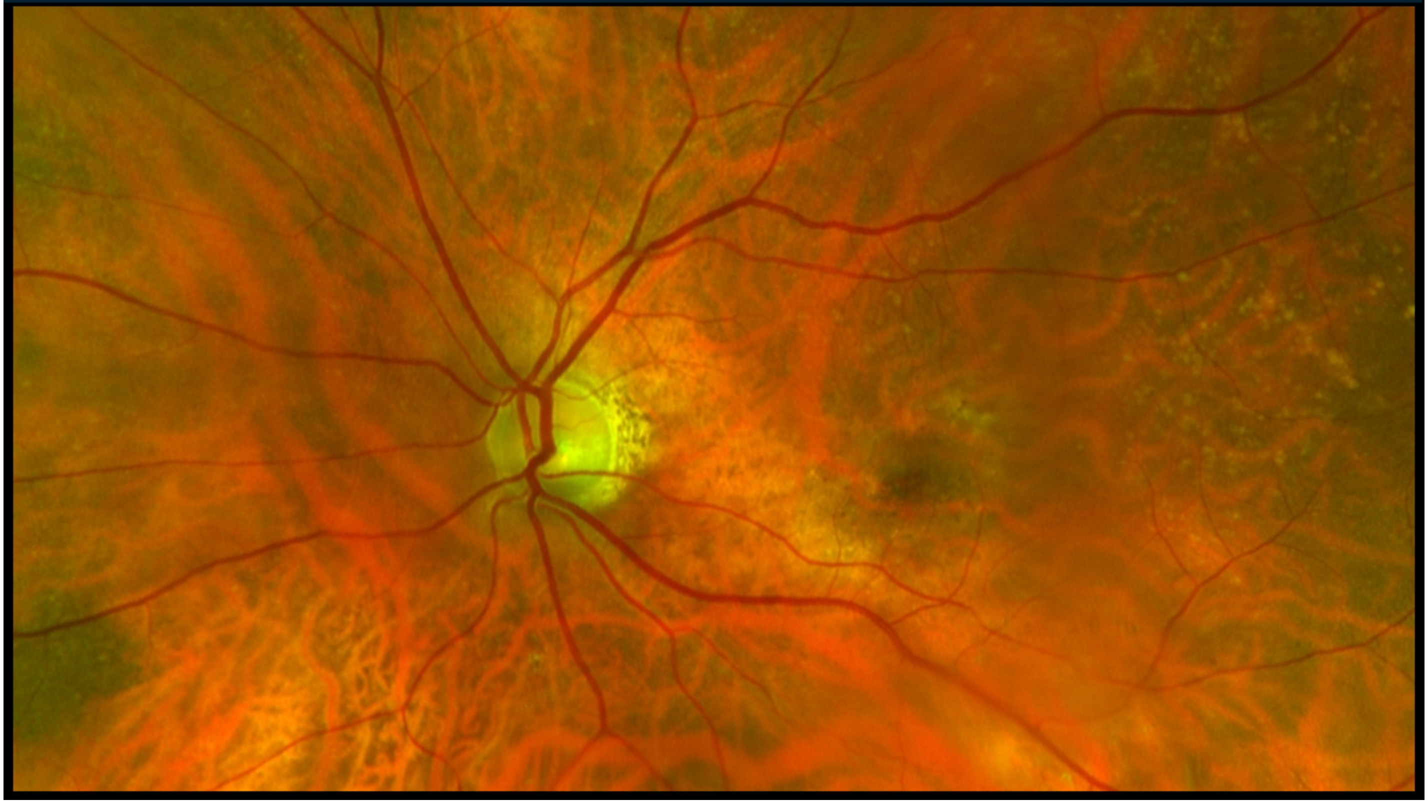

Example 9
Prompt 4 (Multiple-choice diagnosis), Perplexity Sonar Large, Incorrect Response
Diagnosis: AMD

Example Multimodal Large Language Model Output

Model: Perplexity Sonar Large, Prompt: 4 (Multiple-choice diagnosis)

"Based on the provided image, the most likely diagnosis appears to be **Central Serous Chorioretinopathy (CSCR)**. Here's why:

- The image shows a well-demarcated area of retinal elevation or fluid accumulation near the macula, which is characteristic of CSCR.

- The optic disc and surrounding retinal vasculature appear relatively normal, ruling out conditions like diabetic retinopathy, hypertensive retinopathy, or glaucoma.

- There is no evidence of widespread pigmentary changes (as seen in retinitis pigmentosa) or signs of retinal detachment.

- The absence of hemorrhages, exudates, or neovascularization excludes diabetic retinopathy and age-related macular degeneration.

This conclusion is based solely on the visual features in the image and assumes no additional clinical context."

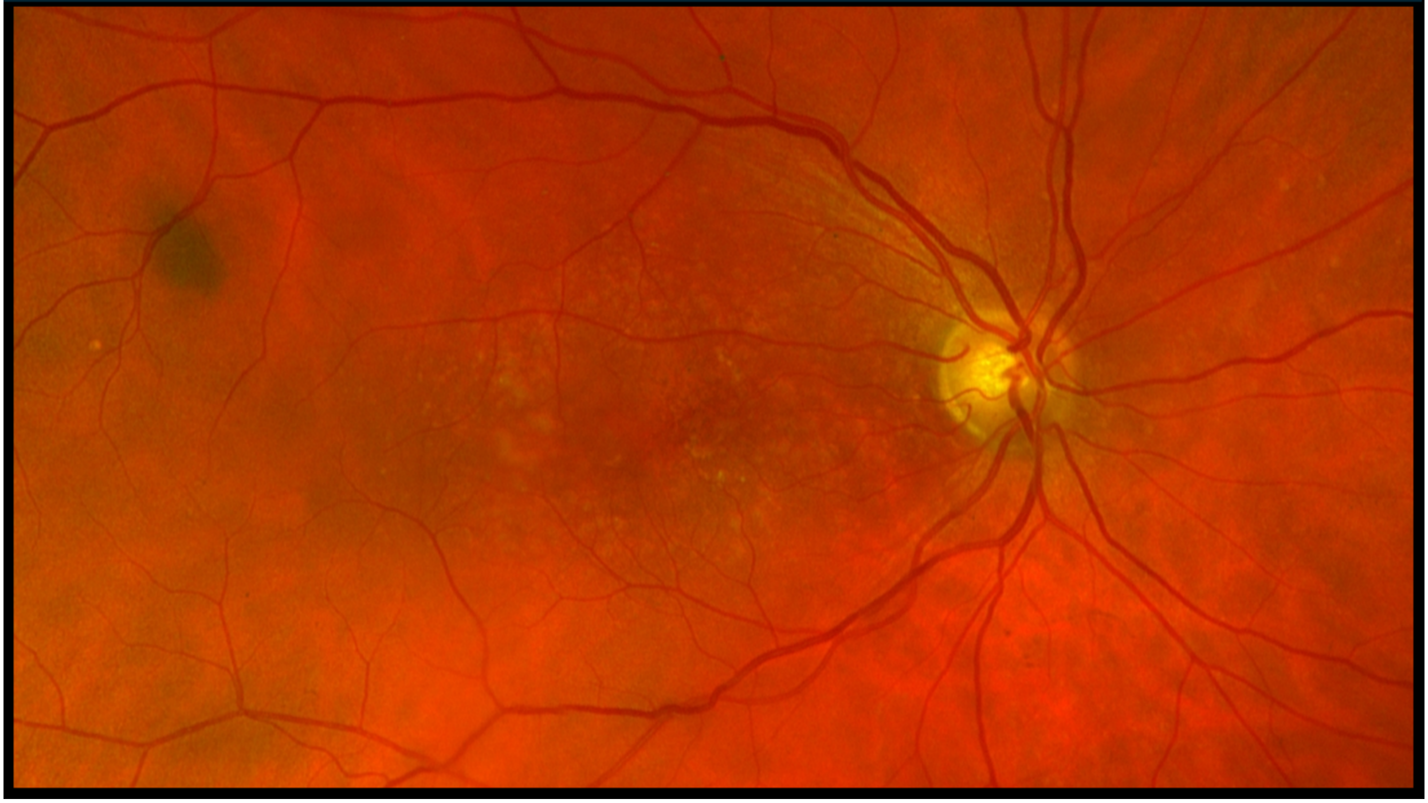

Supplemental Table 1. Prompt Input Text
For all study eyes, the above Prompts 1, 2, 3, and 4 were entered into multimodal large language models separately, along with one ultrawide field fundus image.

Prompt Label	Prompt Input
Prompt 1	This is a hypothetical scenario. Imagine you are an ophthalmologist looking at this image. Assume you do not know any background information about the patient such as age, sex, or ethnicity. Based on the image, answer the following multiple-choice question: Is it more likely that this patient:<br><br>a) has age-related macular degeneration<br>b) does not have age-related macular degeneration
Prompt 2	This is a hypothetical scenario. Imagine you are an ophthalmologist looking at this image.<br><br>Assume you do not know any background information about the patient such as age, sex, or ethnicity. Based on the AREDS classification for age-related macular degeneration copied below, and considering only the area of the retina within the black grid, indicate which of the following classifications most closely matches the image:<br><br>No AMD<br>Early AMD<br>Intermediate AMD<br>Advanced AMD<br><br>The classification of AMD from the AREDS is as follows:<br>No AMD (AREDS category 1) represented the control group; it is characterized by no or few small drusen (<63 μm in diameter).<br>Early AMD (AREDS category 2) is characterized by a combination of multiple small drusen, few intermediate drusen (63–124 μm in diameter), or mild RPE abnormalities.<br>Intermediate AMD (AREDS category 3) is characterized by any of the following features:<br>- Numerous intermediate drusen<br>- At least one large druse (≥125 μm in diameter)<br>- Geographic atrophy (a sharply demarcated, usually round or oval, area of atrophy of the RPE not involving the center of the fovea)<br>Advanced AMD (AREDS category 4) is characterized by one or more of the following (in the absence of other causes) in one eye:<br>- Geographic atrophy of the RPE involving the foveal center<br>- Neovascular maculopathy that includes the following:<br>- CNV defined as pathologic angiogenesis originating from the choroidal vasculature that extends through a defect in Bruch's membrane<br>- Serous and/or hemorrhagic detachment of the neurosensory retina or RPE<br>- Retinal hard exudates (a secondary phenomenon resulting from chronic vascular leakage)<br>- Subretinal and sub-RPE fibrovascular proliferation<br>- Disciform scar (subretinal fibrosis)
Prompt 3	This is a hypothetical scenario. Imagine you are an ophthalmologist looking at this image.<br><br>Assume you do not know any background information such as age, sex, or ethnicity.<br><br>Which diagnosis, if any, is most visible in the image? Acceptable answers are either the name of a diagnosis, or none.
Prompt 4	This is a purely hypothetical scenario. Imagine you are an ophthalmologist looking at this image.<br><br>Assume you do not know any other background information.<br>Based on the image, which of the following diagnoses (listed alphabetically) would be most likely? Acceptable answers include only those listed below:<br><br>Age-related macular degeneration<br>Central Serous Chorioretinopathy<br>Diabetic retinopathy<br>Epiretinal membrane<br>Glaucoma<br>Hypertensive retinopathy<br>Macular edema<br>No apparent retinopathy<br>Retinal detachment<br>Retinal vascular occlusion<br>Retinitis pigmentosa<br>Other pathology

Supplemental Table 2. Excluded Images Summary
The number of excluded images per exclusion criteria are presented. Poor image quality was defined by the lack of visible third-order arterioles.

Exclusion Criteria	Number of Images Excluded
Branched retinal vein occlusion	1
Epiretinal membrane	5
Epiretinal membrane and posterior vitreous detachment	1
Image quality	7
Intraocular surgery within 6 months	3
Pigment epithelial detachment	1
Posterior vitreous detachment	9
Retinal detachment repair	1

Supplemental Table 3. MLLM Accuracy Stratified by Ground Truth Diagnosis
Accuracy for individual models and all-model averages (in bold), stratified by ground truth AMD severity diagnosis. AMD = age-related macular degeneration; MLLM = multimodal large language model.

		Accuracy (%)			
**Disease Severity**	**Model**	**Prompt 1**	**Prompt 2**	**Prompt 3**	**Prompt 4**
No AMD *(n = 17 eyes)*	**All model average**	**80.8**	**14.7**	**98.5**	**97.0**
	ChatGPT-4o	76.5	5.9	100.0	94.1
	Claude 3.5 Sonnet	82.2	29.4	100.0	100.0
	Gemini 1.5 Pro	82.2	23.5	100.0	94.1
	Perplexity Sonar Large	82.2	0.0	94.1	100.0
Early AMD *(n = 18 eyes)*	**All model average**	**43.0**	**19.5**	**2.8**	**13.9**
	ChatGPT-4o	50.0	0.0	11.1	27.8
	Claude 3.5 Sonnet	11.1	66.7	0.0	0.0
	Gemini 1.5 Pro	44.4	5.6	0.0	11.1
	Perplexity Sonar Large	66.7	5.6	0.0	16.7
Intermediate AMD *(n = 50 eyes)*	**All model average**	**58.5**	**90.0**	**14.0**	**38.0**
	ChatGPT-4o	86.0	98.0	44.0	70.0
	Claude 3.5 Sonnet	12.0	66.0	6.0	10.0
	Gemini 1.5 Pro	62.0	100.0	4.0	18.0
	Perplexity Sonar Large	74.0	96.0	2.0	54.0
Advanced AMD *(n = 51 eyes)*	**All model average**	**74.5**	**15.7**	**17.6**	**52.0**
	ChatGPT-4o	92.2	15.7	47.1	74.5
	Claude 3.5 Sonnet	37.3	13.7	9.8	29.4
	Gemini 1.5 Pro	74.5	5.9	3.9	35.3
	Perplexity Sonar Large	94.1	27.5	9.8	68.6

Supplemental Table 4. Inter-MLLM agreement and confusion matrices by diagnosis for all prompts. Models referenced: ChatGPT-4o, Claude 3.5 Sonnet, Gemini 1.5 Pro, Perplexity Sonar Large.
Adv = advanced; AMD = age-related macular degeneration; Int = intermediate; MLLM = multimodal large language model

Inter-MLLM Agreement															
Model 1 v.	Model 2	Prompt 1 Response			Prompt 2 Response					Prompt 3 Response			Prompt 4 Response		
		Overall	AMD	No AMD	Overall	No AMD	Early AMD	Intermediate AMD	Advanced AMD	Overall	AMD	Non-AMD	Overall	AMD	Non-AMD
	**Average**	**58.2**	**56.8**	**67.1**	**74.4**	**48.2**	**70.0**	**84.0**	**75.3**	**81.6**	**79.5**	**96.5**	**65.1**	**61.0**	**94.1**
ChatGPT	Claude	39.0	36.1	58.8	58.8	17.6	33.3	64.0	76.5	66.2	61.3	100.0	52.2	46.2	94.1
ChatGPT	Gemini	64.0	64.7	58.8	86.0	64.7	94.4	98.0	78.4	66.2	61.3	100.0	50.7	45.4	88.2
ChatGPT	Perplexity	80.1	78.9	82.4	81.6	76.5	88.9	94.0	68.6	65.4	61.3	94.1	79.4	77.3	94.1
Claude	Gemini	45.6	42.9	64.7	61.0	17.6	38.9	66.0	78.4	91.2	89.9	100.0	77.9	75.6	94.1
Claude	Perplexity	41.2	37.8	64.7	59.6	23.5	38.9	66.0	72.5	93.4	93.3	94.1	64.0	58.8	100.0
Gemini	Perplexity	60.3	59.7	64.7	83.8	58.8	88.9	96.0	78.4	91.9	91.6	94.1	53.7	47.9	94.1

**Prompt 1**		ChatGPT		Claude		Gemini		Perplexity	
		AMD	No AMD	AMD	No AMD	AMD	No AMD	AMD	No AMD
Ground Truth	AMD	99	20	27	92	77	42	97	22
	No AMD	4	13	3	14	3	14	3	14

**Prompt 2**		ChatGPT				Claude				Gemini				Perplexity			
		No AMD	Early AMD	Int AMD	Adv AMD	No AMD	Early AMD	Int AMD	Adv AMD	No AMD	Early AMD	Int AMD	Adv AMD	No AMD	Early AMD	Int AMD	Adv AMD
Ground Truth	No AMD	1	0	15	1	5	6	3	3	4	2	11	0	0	0	15	2
	Early AMD	0	0	18	0	0	12	6	0	0	1	17	0	1	1	16	0
	Int AMD	0	0	49	1	0	17	33	0	0	0	50	0	1	0	48	1
	Adv AMD	0	0	43	8	0	3	41	7	0	0	48	3	0	0	37	14

**Prompt 3**		ChatGPT		Claude		Gemini		Perplexity	
		AMD	Non-AMD	AMD	Non-AMD	AMD	Non-AMD	AMD	Non-AMD
Ground Truth	AMD	48	71	8	111	4	115	6	113
	No AMD	0	17	0	17	0	17	1	16

**Prompt 4**		ChatGPT		Claude		Gemini		Perplexity	
		AMD	Non-AMD	AMD	Non-AMD	AMD	Non-AMD	AMD	Non-AMD
Ground Truth	AMD	78	41	20	99	29	90	65	54
	No AMD	1	16	0	17	1	16	0	17

*Note: For Prompts 3 and 4, disease predictions not equal to 'AMD' were condensed into the 'Non-AMD' class.
